# Supplementary material for: Vascular endothelium deploys caveolin-1 to regulate oligodendrogenesis after chronic cerebral ischemia in mice
Source: Nat Commun. 2022 Nov 10;13:6813. doi: 10.1038/s41467-022-34293-7 (PMC9649811; doi:10.1038/s41467-022-34293-7)
Supplement: Supplementary file 2 — Supplementary Information [file 41467_2022_34293_MOESM2_ESM.pdf]

Supplementary Materials for

Vascular endothelium deploys caveolin-1 to regulate oligodendrogenesis after chronic cerebral ischemia in mice

Ying Zhao†, Wusheng Zhu†, Ting Wan†, Xiaohao Zhang†, Yunzi Li, Zhenqian Huang, Pengfei Xu, Kangmo Huang, Ruidong Ye*✉, Yi Xie*✉, Xinfeng Liu*✉

†Contributed equally.

*Corresponding author. ✉email: xfliu2@vip.163.com (X.L.) or xy_307@126.com (Y.X.) or yeruid@gmail.com (R.Y.)

The file includes:

Fig. S1. Time-course changes of demyelination in the CC after BCAS.

Fig. S2. Time-course changes of endothelial impairments in the CC after BCAS.

Fig. S3. The co-immunoprecipitation and co-immunostaining of Cav-1 and HSP90α in BMECs.

Fig. S4. Cav-1 is predominantly expressed in BMECs.

Fig. S5. The alterations of capillary and arteriolar caveolae in hypoxia.

Fig. S6. Fluorescent FISH of *Hsp90α* mRNA with different cell markers in the CC.

Fig. S7. Endothelial Cav-1 was not altered in HSP90α siRNA-treated BCAS mice of 2 genotypes.

Fig. S8. PEI-antagomir relieves endothelial dysfunction *in vivo*.

Fig. S9. Nanoparticle-antagomir attenuates ischemic demyelination via Cav-1.

Fig. S10. Nanoparticle-antagomir improves cognitive function via Cav-1.

Table S1. Comparison of baseline data according to patients with and without leukoaraiosis.

Table S2. Primers for reverse transcription.

Table S3. Real-time PCR primers in this study.

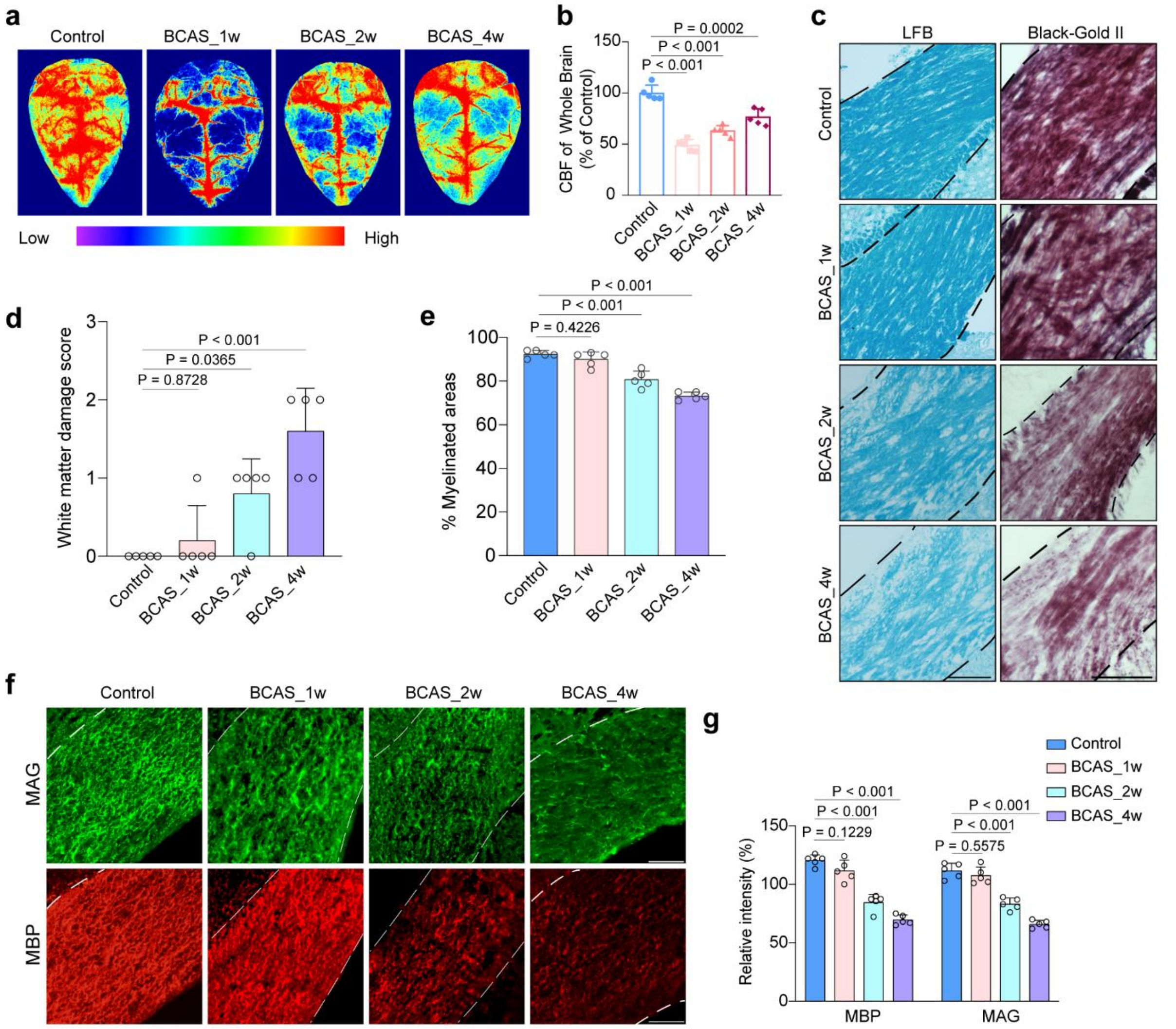

Figure S1. Time-course changes of demyelination in the CC after BCAS.
(**a,b**) Representative CBF images and quantitative analysis of control, BCAS_1w, BCAS_2w, and BCAS_4w mice (n = 5 mice/group). (**c**) Representative Luxol fast blue and black-gold II staining (n = 5 mice/group). Scale bar, 20 μm. (**d,e**) Quantitative analysis of white matter damage severity and myelinated area in 4 groups (n = 5 mice/group). (**f,g**) Representative images and quantifications of MAG and MBP in 4 groups (n = 5 mice/group). Scale bar, 20 μm. All data are presented as the mean ± SD. The data were compared by one-way ANOVA with Tukey post hoc test. Source data are provided as a Source Data file.

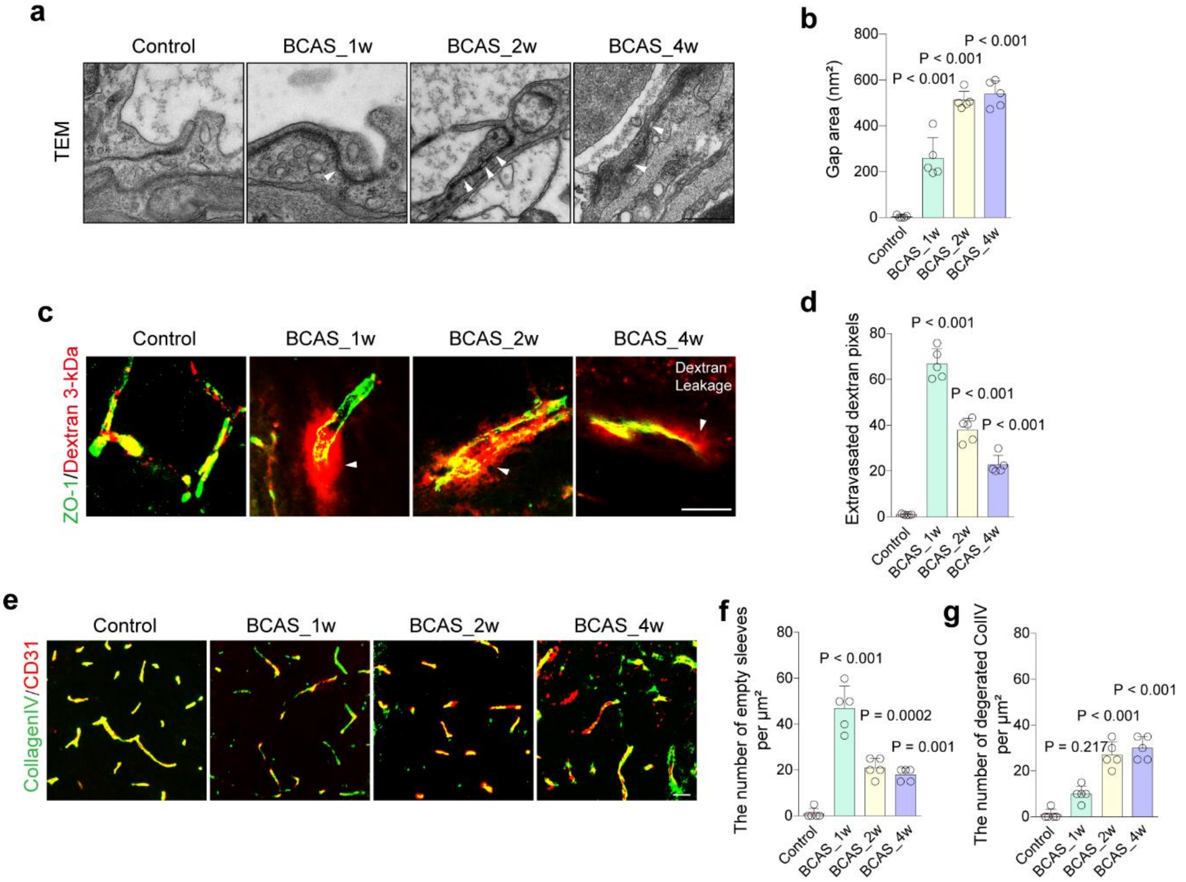

Figure S2. Time-course changes of endothelial impairments in the CC after BCAS.

(**a, b**) Representative TEM images showing tight junction gaps (white arrow) between endothelial cells (n = 5 mice/group). Scale bar, 500 nm. (**c, d**) Representative images and the quantification for ZO-1/dextran co-staining. White arrows indicated the extravasated dextran (n = 5 mice/group). Scale bar, 20 μm. (**e–g**) Representative images of collagen IV and CD31 staining images. Quantifications were for the number of basement membrane sleeves devoid of endothelial cells and the number of basement membranes devoid of collagen IV (n = 5 mice/group). Scale bar, 20 μm. All data are presented as the mean ± SD. The data were compared by one-way ANOVA with Tukey post hoc test. Source data are provided as a Source Data file.

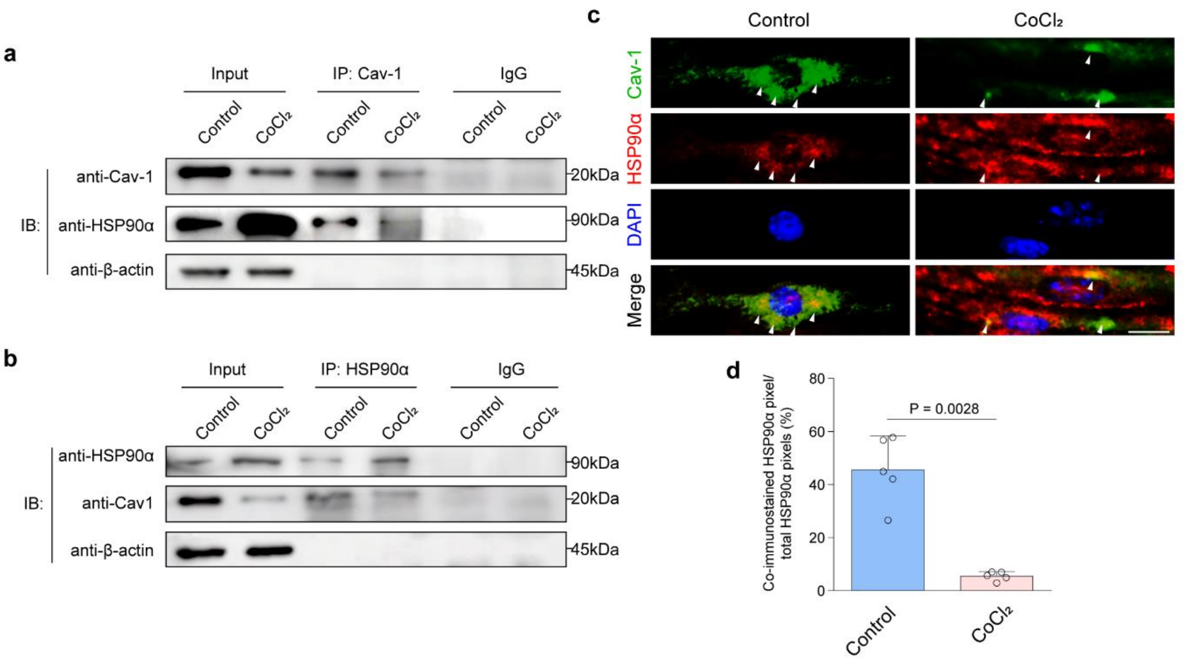

Figure S3. The co-immunoprecipitation and co-immunostaining of Cav-1 and HSP90α in BMECs. (**a, b**) After immunoprecipitation with anti-Cav-1 or anti-HSP90α antibodies respectively, the immunoprecipitates were analyzed by immunoblotting with anti-Cav-1 and anti-HSP90α antibodies (n = 5 replicates). (**c, d**) Representative co-immunostaining images and quantification of Cav-1 (green) and HSP90α (red) in endothelial cells after $CoCl_2$ treatment. White arrows indicated the co-localization of Cav-1 and HSP90α (n = 5 independent primary cell cultures/group). Scale bar, 20 μm. All data are presented as the mean ± SD. The data were compared by paired t-test. Source data are provided as a Source Data file.

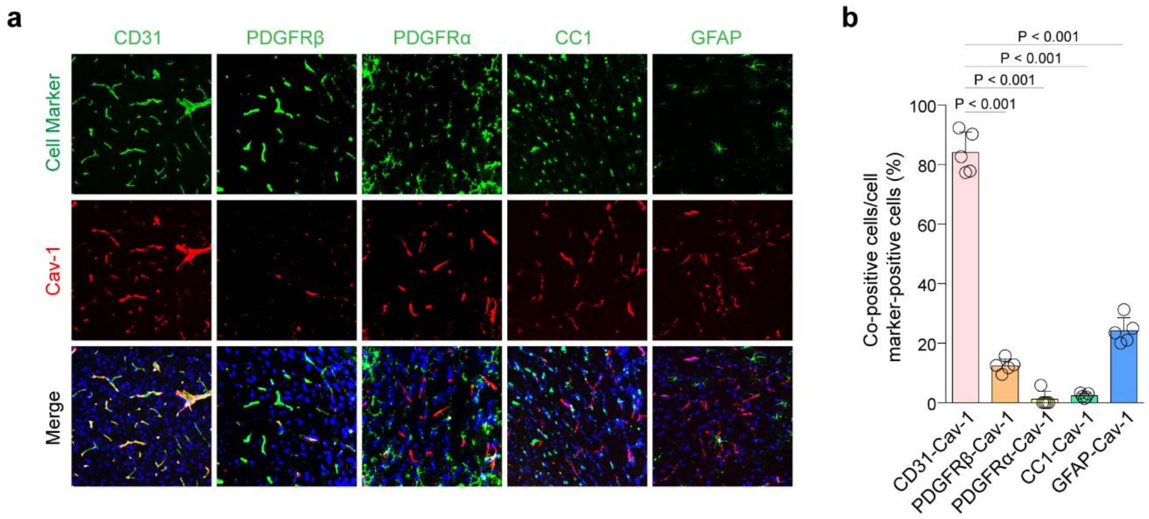

Figure S4. Cav-1 is predominantly expressed in BMECs.

(**a, b**) Double immunostaining and the quantification of Cav-1 with CD31 (endothelial marker), PDGFRβ (pericyte marker), PDGFRα (OPC marker), CC1 (mature oligodendrocyte marker), and GFAP (astrocyte marker) (n = 5 independent primary cell cultures/group) Scale bar, 20 μm. The data are presented as mean ± SD. Data were compared by one-way ANOVA with Tukey post hoc test. Source data are provided as a Source Data file.

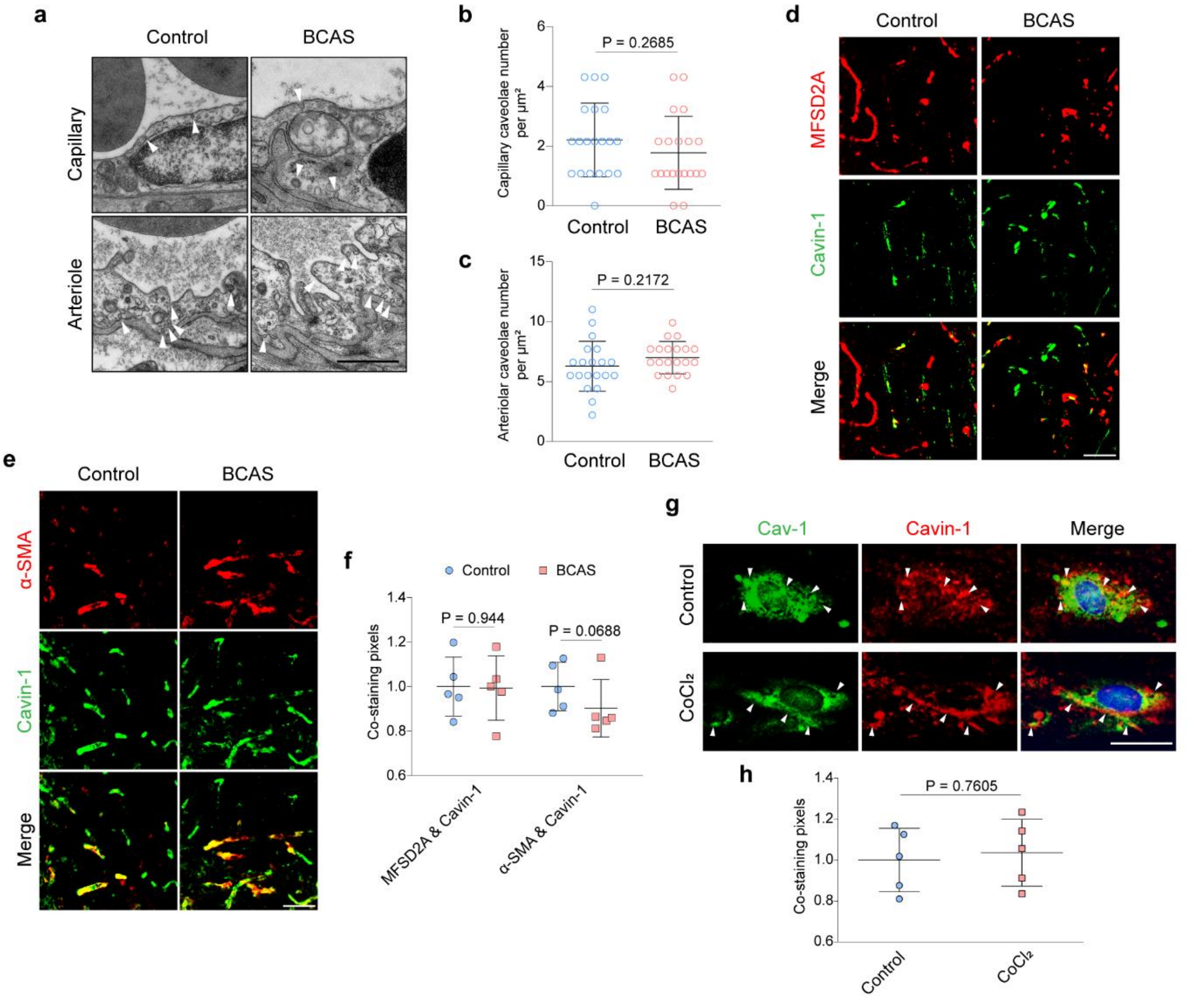

Figure S5. The alterations of capillary and arteriolar caveolae in hypoxia. (**a–c**) Representative TEM images of vascular caveolae (white arrow) and statistical analyses of caveolae number (n = 5 mice, 4 capillaries or arterioles in each sample). Scale bar, 1 μm. (**d–f**) Representative Cavin-1 immunostaining with capillary and arteriolar marker, MFSD2A and α-SMA, and their pixel ratios (n = 5 mice/group). Scale bar, 20 μm. (**g, h**) *In vitro* staining and quantification of co-localized Cav-1 and Cavin-1 in cultured BMECs. The white arrows indicated the co-localization of the two proteins (n = 5 independent primary cell cultures/group). Scale bar, 20 μm. All data are presented as the mean ± SD. The data were compared by paired t-test. Source data are provided as a Source Data file.

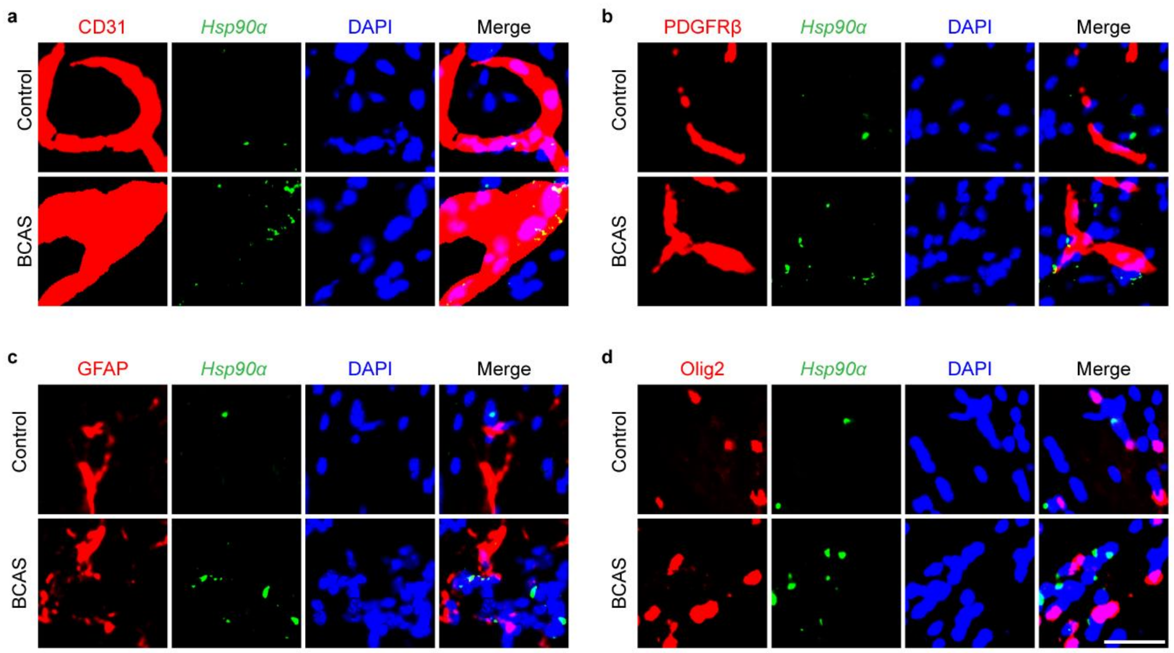

Figure S6. Fluorescent FISH of *Hsp90α* mRNA with different cell markers in the CC. n = 5 mice/group. Scale bar, 20 μm.

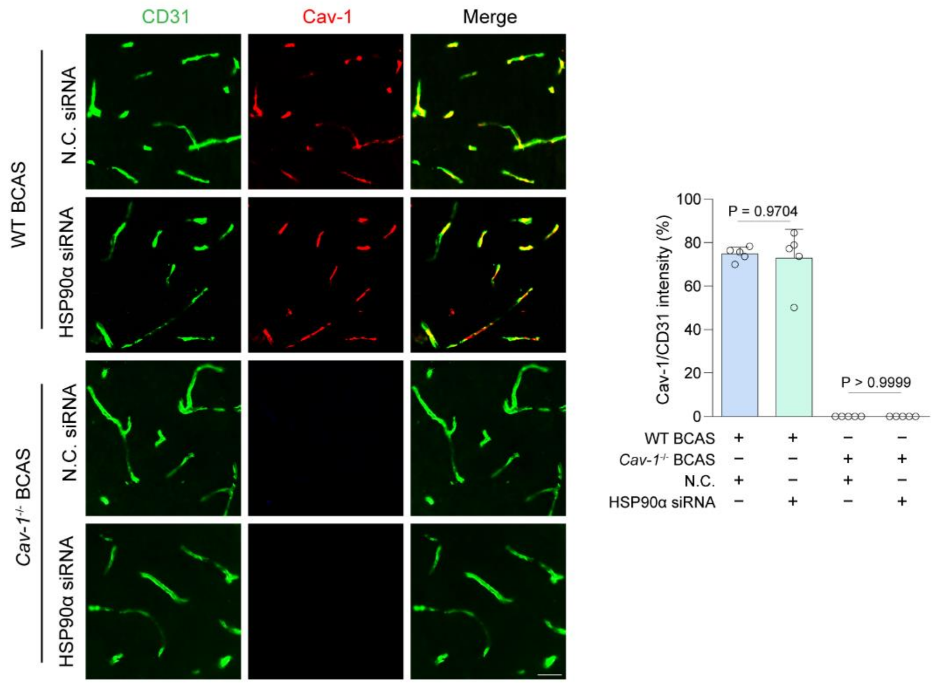

Figure S7. Endothelial Cav-1 was not altered in HSP90α siRNA-treated BCAS mice of 2 genotypes. Representative immunofluorescence images and quantification of CD31 with Cav-1. n = 5 mice/group. Scale bar, 20 μm. The data are presented as mean ± SD. Data were compared by one-way ANOVA with Tukey post hoc test. Source data are provided as a Source Data file.

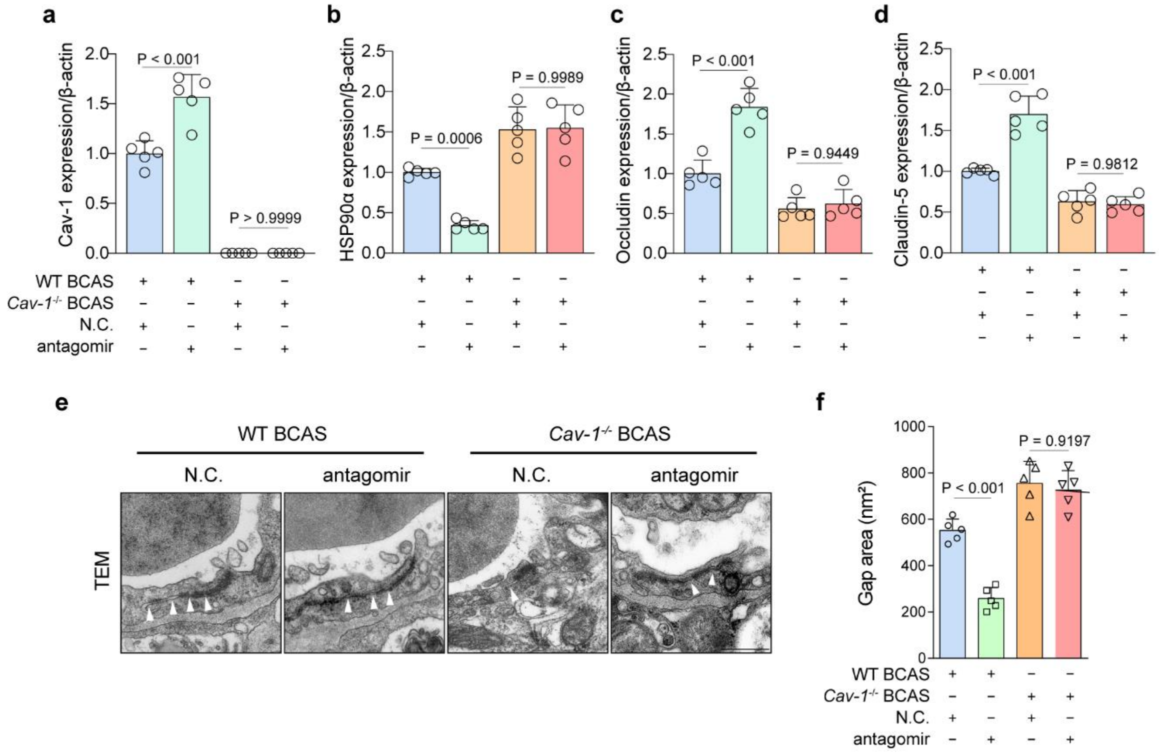

Figure S8. PEI-antagomir relieves endothelial dysfunction *in vivo*.

(**a–d**) Qualifications for western blotting of Cav-1, HSP90α, Occludin, and Claudin-5 in BCAS mice of 2 genotypes treated with N.C. or antagomir (n = 5 mice/gourp). (**e, f**) Representative ultrastructural images and quantifications showing the differences in tight junction gaps (white arrow) between endothelial cells in CC from mice of 4 groups (n = 5 mice/group). Scale bar, 500 nm. All data are presented as the mean ± SD. The data were compared by one-way ANOVA with Tukey post hoc test. Source data are provided as a Source Data file.

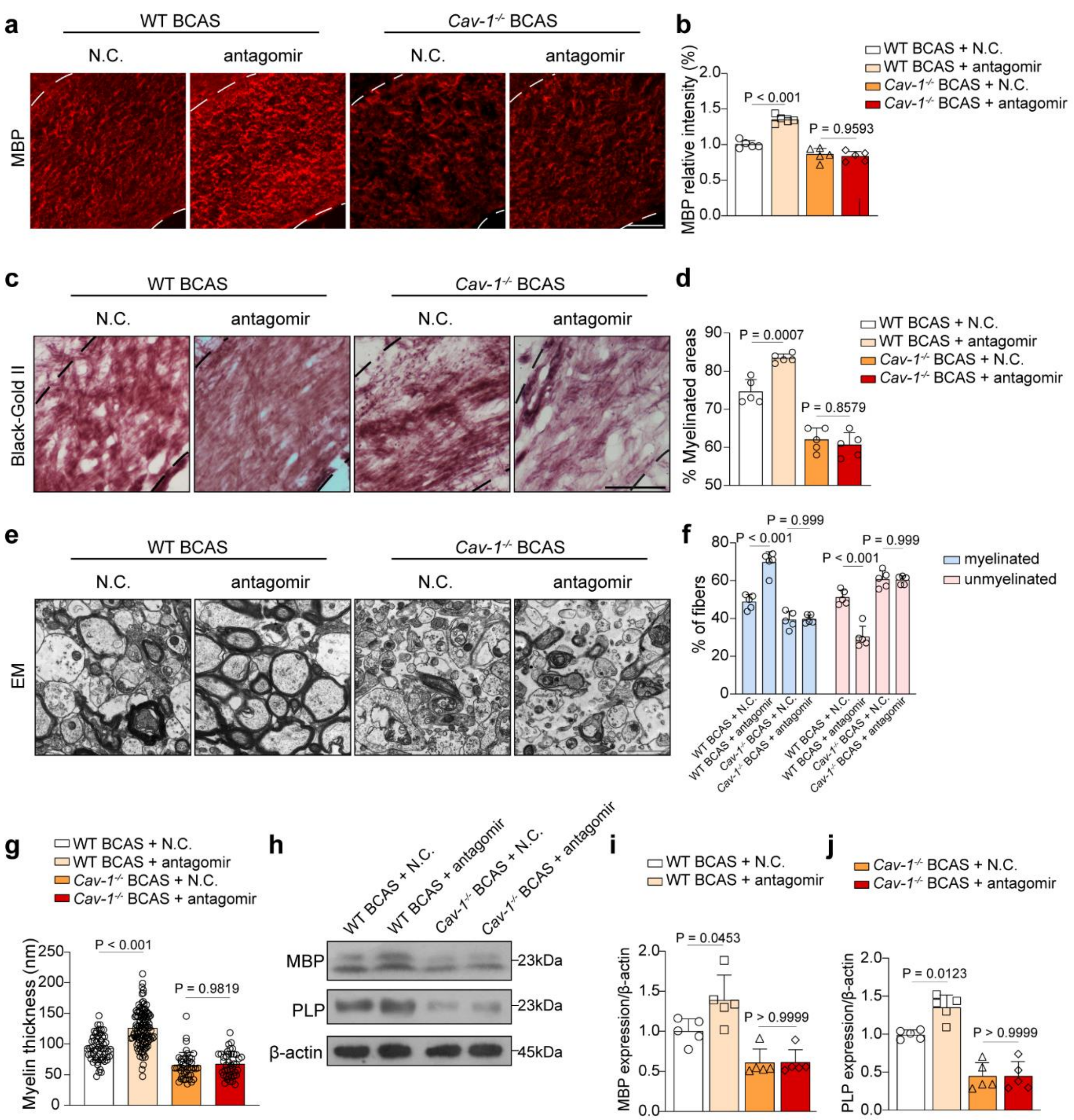

Figure S9. Nanoparticle-antagomir attenuates ischemic demyelination via Cav-1. (**a–d**) Representative MBP, black gold-II staining, and their quantifications (n = 5 mice/group). Scale bar, 50 µm. (**e–g**) Representative ultrastructural images and quantitative analyses of myelinated fibers and myelin thickness (n = 5 mice/group). Scale bar, 2 µm. (**h–j**) Western blotting and statistical analyses showing the expression of myelin proteins, MBP and PLP. Results represent the mean for the relative band intensity of five replicates. All data are presented as the mean ± SD. The data were compared by one-way ANOVA with Tukey post hoc test. Source data are provided as a Source Data file.

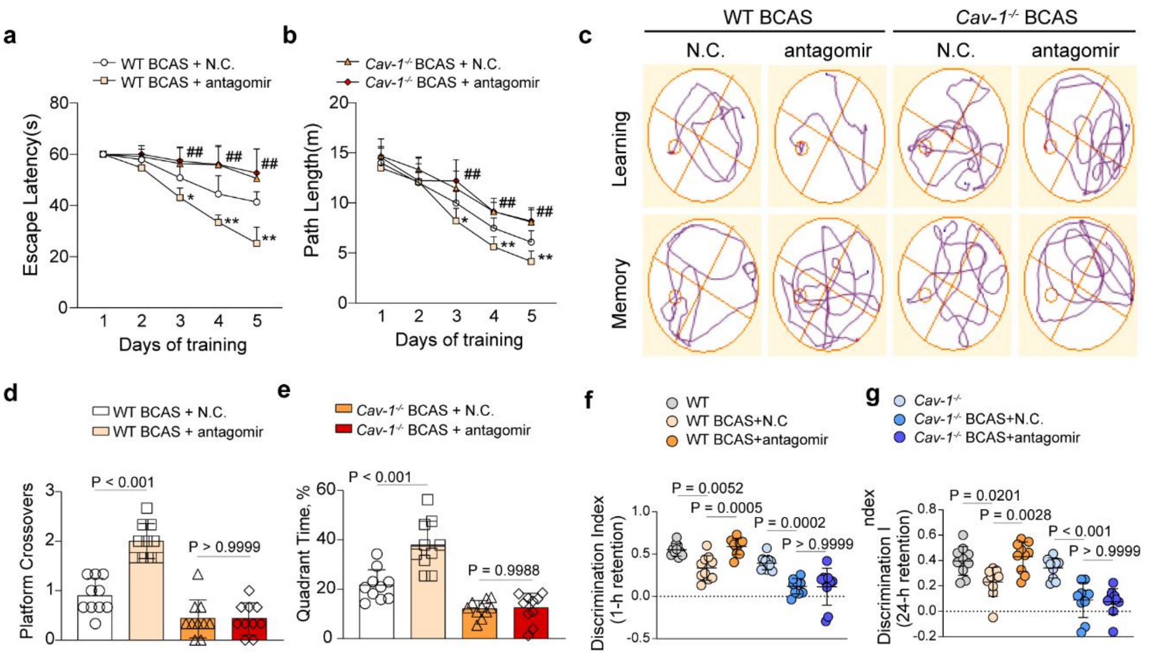

Figure S10. Nanoparticle-antagomir improves cognitive function via Cav-1.

(**a, b**) The escape latency and path length recorded in the duration of learning stage (n = 10 mice/group; mean ± SD.; **P* < 0.05, ***P* < 0.01 *vs.* WT BCAS + N.C. and ##*P* < 0.01 *vs.* WT BCAS + antagomir by two-way repeated-measures ANOVA with Tukey post hoc test). (**c**) Representative traces of swimming path in hidden platform test (top panel, "learning") and probe trial stage (bottom panel, "memory") (n = 10 mice/group). (**d, e**) The crossovers and percentage of time spent in the platform quadrant in the probe trial (n = 10 mice/group). (**f, g**) The discrimination index for the novel object in short-term or long-term memory test (n = 10 mice/group). All data are presented as the mean ± SD. The data were compared by two-way repeated-measures ANOVA with Tukey post hoc test. Source data are provided as a Source Data file.

Table S1. Comparison of baseline data according to patients with and without leukoaraiosis.

Variable	With leukoaraiosis, n = 88	Without leukoaraiosis, n = 66	*P* value
Demographic characteristics			
Age, years	69.4 ± 9.0	63.9 ± 9.3	0.001
Male, n (%)	46 (52.3)	38 (57.6)	0.513
Body mass index, kg/m^2	23.3 ± 3.0	23.0 ± 2.7	0.458
Vascular risk factor, n (%)			
Hypertension	64 (72.7)	41 (62.1)	0.162
Diabetes mellitus	31 (35.2)	10 (15.2)	0.005
Hyperlipidemia	12 (13.6)	10 (15.2)	0.790
Smoking	44 (50.0)	26 (39.4)	0.191
Laboratory data			
Hsa-miR-3074-3p	70.179 (1.490, 170.619)	0.495 (0.160, 6.279)	0.001
Caveolin-1, ng/ml	0.117 (0.043, 0.217)	0.389 (0.269, 0.380)	0.001

Table S2. Primers for reverse transcription.

Primer name	Primer sequence
miR-3074-1-3p	CCTGTTGTCTCCAGCCACAAAAGAGCACAATATTTCAGGAGACAACAGGCGGTGCC
miR-34b-5p	CCTGTTGTCTCCAGCCACAAAAGAGCACAATATTTCAGGAGACAACAGGACAATCA
miR-34c-5p	CCTGTTGTCTCCAGCCACAAAAGAGCACAATATTTCAGGAGACAACAGGGCAATCA
miR-204-5p	CCTGTTGTCTCCAGCCACAAAAGAGCACAATATTTCAGGAGACAACAGGAGGCATA
miR-96-5p	CCTGTTGTCTCCAGCCACAAAAGAGCACAATATTTCAGGAGACAACAGGAGCAAAA
miR-466a/e-3p	CCTGTTGTCTCCAGCCACAAAAGAGCACAATATTTCAGGAGACAACAGGTCTTATG
miR-211-5p	CCTGTTGTCTCCAGCCACAAAAGAGCACAATATTTCAGGAGACAACAGGAGGCAAA
miR-448-3p	CCTGTTGTCTCCAGCCACAAAAGAGCACAATATTTCAGGAGACAACAGGATGGGAC
miR-1264-3p	CCTGTTGTCTCCAGCCACAAAAGAGCACAATATTTCAGGAGACAACAGGACAGGTG
miR-466b/c/p-3p	CCTGTTGTCTCCAGCCACAAAAGAGCACAATATTTCAGGAGACAACAGGTCTTATG
hsa-miR-3074-3p	CCTGTTGTCTCCAGCCACAAAAGAGCACAATATTTCAGGAGACAACAGGAGGTGCC
cel-miR-39-3p	CCTGTTGTCTCCAGCCACAAAAGAGCACAATATTTCAGGAGACAACAGGCAAGCTG

Table S3. Real-time PCR primers in this study.

Primer name	Primer sequence	
miR-3074-1-3p	Forward	CGGGCGATATCAGCTCAGTA
	Reverse	CAGCCACAAAAGAGCACAAT
miR-34b-5p	Forward	GCGGCAGGCAGTGTAATTAGC
	Reverse	CAGCCACAAAAGAGCACAAT
miR-34c-5p	Forward	GCGGCAGGCAGTGTAGTTAGC
	Reverse	CAGCCACAAAAGAGCACAAT
miR-204-5p	Forward	CGGGCTTCCCTTTGTCATCC
	Reverse	CAGCCACAAAAGAGCACAAT
miR-96-5p	Forward	CGCCGTTTGGCACTAGCACAT
	Reverse	CAGCCACAAAAGAGCACAAT
miR-466a/e-3p	Forward	GCGGCTATACATACACGCACA
	Reverse	CAGCCACAAAAGAGCACAAT
miR-211-5p	Forward	CGGGCTTCCCTTTGTCATCC
	Reverse	CAGCCACAAAAGAGCACAAT
miR-448-3p	Forward	CGCCGTTGCATATGTAGGAT
	Reverse	CAGCCACAAAAGAGCACAAT
miR-1264-3p	Forward	GCGGCCAAATCTTATTTGAG
	Reverse	CAGCCACAAAAGAGCACAAT
miR-466b/c/p-3p	Forward	GCGGCATACATACACGCACA
	Reverse	CAGCCACAAAAGAGCACAAT
Caveolin-1	Forward	GAAGGGACACACAGTTTCG
	Reverse	AGGAAGGAGAGAATGGCAA
HIF-1α	Forward	GATGACGGCGACATGGTTTAC
	Reverse	CTCACTGGGCCATTTCTGTGT
HIF-2α	Forward	TCCTTCGGACACATAAGCTCC
	Reverse	GACAGAAAGATCATGTCACCGT
PDK-1	Forward	GGACTTCGGGTCAGTGAATGC
	Reverse	TCCTGAGAAGATTGTCGGGGA
BNIP3	Forward	CTGGGTAGAACTGCACTTCAG
	Reverse	GGAGCTACTTCGTCCAGATTCAT
VEGF	Forward	GCACATAGAGAGAATGAGCTTCC
	Reverse	CTCCGCTCTGAACAAGGCT
U6	Forward	CTCGCTTCGGCAGCACATATACT
	Reverse	ACGCTTCACGAATTTGCGTGTC
GAPDH	Forward	AAGAAGGTGGTGAAGCAGG
	Reverse	GAAGGTGGAAGAGTGGGAGT
hsa-miR-3074-3p	Forward	CGGGCGATATCAGCTCAGTA
	Reverse	CAGCCACAAAAGAGCACAAT
cel-miR-39-3p	Forward	GCGCTCACCGGGTGTAAAT
	Reverse	CAGCCACAAAAGAGCACAAT
